# Frictional Resistance and Surface Roughness of Reduced Graphene Oxide Coated Stainless‐Steel Archwires: An In Vitro Study

**DOI:** 10.1155/ijod/7815889

**Published:** 2026-08-11

**Authors:** P. Shreeya Varma, Arun S. Urala, Ramya Vijeta Jathanna, Suresh D. Kulkarni

**Affiliations:** ^1^ Department of Orthodontics and Dentofacial Orthopedics, Manipal College of Dental Sciences, Manipal Academy of Higher Education, Manipal, India, manipal.edu; ^2^ Manipal Institute of Applied Physics, Manipal Academy of Higher Education, Manipal, India, manipal.edu

**Keywords:** coating, frictional resistance, nanoparticles, orthodontic archwires, surface characterization

## Abstract

**Objectives:**

The objective of this study was to evaluate the effect of reduced graphene oxide (rGO) coating on the frictional resistance and surface characteristics of stainless‐steel (SS) orthodontic archwires compared with uncoated SS wires (SSWs).

**Materials and Methods:**

This in vitro study is designed to evaluate the frictional resistance and surface characterization of a coating. Twenty 0.019 × 0.025‐inch SS archwires were divided into two groups (*n* = 10). Experimental wires were coated with rGO using an electrochemical deposition method. Frictional resistance was evaluated using a universal testing machine, while coating morphology and surface characteristics were assessed using Raman spectroscopy, field emission scanning electron microscopy (FESEM), optical microscopy, and EDX analysis.

**Results:**

Analysis via FESEM confirmed the thickness and uniformity of the coating. Archwires coated with rGO showed reduced frictional resistance against the uncoated archwires. The reduction was statistically significant under normal load and stress situations.

**Conclusion:**

In this study, the use of rGO coating markedly decreased the frictional resistance of SS orthodontic archwires in comparison to uncoated wires. The surface characterization confirmed the structural integrity of the rGO covering, thereby facilitating better interlayer sliding and improving tribological performance.

## 1. Introduction

Carbon‐based nanomaterials are widely utilized across numerous scientific disciplines owing to their unique physiochemical properties and versatile structural configurations [1, 2]. Among these materials, graphene, a material composed of single crystalline carbon arranged in a honeycomb structure, is among the strongest and thinnest substances. Its derivatives exhibit exceptional mechanical, chemical, and thermal properties, along with a high surface area and antimicrobial characteristics. And remarkable tribological behavior [3]. Graphene oxide is produced by the oxidation of graphite, introducing oxygen‐containing functional groups that improve its dispersibility and facilitate further chemical modifications. Subsequent reduction of graphene oxide yields reduced graphene oxide (rGO), which partially restores the graphitic structure while retaining functional groups that improve the adhesion to metallic substrates. Owing to these properties, graphene‐based nanomaterials have found promising applications in tissue engineering, drug delivery, implant surface modifications, restorative dentistry, and orthodontic biomaterials [4, 5]. Furthermore, recent investigations have demonstrated that nano‐graphene oxide can enhance the mechanical properties and antibiofilm activity of dental materials, highlighting its potential for orthodontic applications [6].

Orthodontic archwires produce biomechanical forces that are passed through the bracket interface to aid in tooth alterations. These forces are very important in clinical practice. Light, sustained forces are excellent for regulated tooth movement within physiological boundaries, hence reducing hyalinization, resorption, and patient discomfort [7]. However, when an archwire slides through the bracket slot, static and kinetic friction are generated, contributing significantly to the resistance to sliding and affecting the efficiency of tooth movement [8].

Numerous variables influence the frictional resistance, including the type of the bracket, the ligating force, the angle of the archwire to the bracket, the size and shape of the slot and wire, the surface characteristics, and environmental conditions like dryness and deionization [9].

Also, rectangular wires, owing to their larger contact area with the bracket slots, generally exhibit greater frictional resistance than round wires [10].

Stainless‐steel (SS) archwires have retained popularity in orthodontics due to their favorable mechanical properties, dimensional stability, corrosion resistance, and ability to deliver precise tooth movement, including space closures and finishing procedures [11]. An elevation in surface roughness can result in enhanced friction, particularly under dry or boundary‐lubricated conditions, as rougher surfaces possess a larger contact area and generate more asperity interactions, leading to more resistance to sliding. [12]

Over the past two decades, numerous efforts have been made to reduce frictional resistance between archwires and brackets using various forms of nanocoating. The surface coating approach is identified as one of the most effective methods for modifying the friction and wear characteristics of archwire‐bracket contact systems [13].

The objective of this research is to enhance the friction and wear characteristics, as well as the surface properties, of the archwire‐bracket sliding contacts by applying rGO over commercial SS archwires.

## 2. Materials and Methodology

Before commencing the study, approval was obtained from the Institutional Ethics Committee (IEC2: 336/2024). The sample size was calculated using G‐Power software with a power of 80%, an α‐error of 5%, an effect size of 1.4, and two groups, resulting in a required sample size of 10 per group. All specimens were prepared, coated, and tested using identical experimental conditions to minimize variability. Blinding was not performed because the coating was visually identifiable during specimen preparation and testing.

### 2.1. Preparation of Wire Samples in the Experimental Group

Approximately 0.019 × 0.025 inch straight rectangular SS archwires of a sample size of 10 were used. rGO powder (purity >98%; thickness‐0.5–2 nm) was procured commercially from Nano Research Lab. rGO powder (1 mg/mL) was dispersed in deionized (DI) water by ultrasonication for 90 min to achieve a homogeneous suspension. SS wires (SSWs) were sequentially cleaned in acetone, isopropyl alcohol, and ethanol using ultrasonication for 10 min in each solvent, followed by drying in an oven at 80°C for 10 min. The cleaned SSW was used as the working electrode (anode), while platinum served as the counter electrode (cathode). Both electrodes were immersed in the prepared rGO suspension for 30 min to ensure sufficient wettability. Subsequently, a constant potential of 10 V was applied across the electrodes using a potentiostat (Auto Lab Compact) for 8 min under moderate stirring. Successful deposition of rGO on the SSW was indicated by a visible color change from metallic gray to golden yellow. Under the proper condition, particles aggregate to form the layered film that adheres to the surface of SS. The coated sample was rinsed with DI water and dried in an oven at 80°C for 2 h. To get a rGO coating on a lesser thickness, the above procedure was repeated with the same applied potential for 4 mins. The electrochemical deposition protocol was adapted from previously described methods for graphene deposition on SS substrates [14].

### 2.2. Verification of rGO Coating

All specimens were tested under identical experimental conditions using the same universal testing machine, bracket assembly, cross‐head speed (10 mm/min), and loading protocol. No additional load or stress was intentionally applied to either group. The stress (MPa) values reported in the study were calculated from the measured frictional force generated during sliding and therefore represent the mechanical response of the archwire‐bracket system rather than differences in the externally applied loading conditions. Prior to frictional testing, successful deposition of rGO on the SS archwires was confirmed using multiple characterization techniques. Optical microscopy was initially used to verify the uniformity and continuity of the deposited coating. Raman spectroscopy was subsequently performed to identify the characteristic D (~1350 cm^−1^) and G (~1590 cm^−1^) bands of rGO, confirming its successful deposition on the wire surface. field emission scanning electron microscopy (FESEM; Carl Zeiss Sigma 300, Carl Zeiss AG, Germany) was employed to evaluate coating morphology, continuity, and approximate thickness, while energy dispersive X‐ray spectroscopy (EDS) elemental mapping confirmed the presence and distribution of carbon and oxygen on the coated surface. Images were obtained under high‐vacuum mode using an accelerating voltage of 5–15 kV and a working distance of ~8–10 mm. Surface morphology was assessed at multiple magnifications ranging from 500x to 50,000x. Only specimens demonstrating a continuous and uniform rGO coating were included for frictional testing.

### 2.3. Archwire Surface Characterization Evaluation

The surface characterization of both coated and uncoated SS (i.e., Group A and B) archwires, following immersion in artificial saliva, was assessed using FESEM. Each selected wire sample was truncated to a length of 1 cm, utilizing a distal end cutter. Scanning was conducted in the air at a rate of 10 Hz. Each group comprised 10 specimens to assess surface roughness, with each specimen yielding three measurements within a 10 × 10 µm area. The software provided with the FESEM was used to evaluate the wire’s external surface. The Ra, Rq, and Rmax values were obtained using the software. The recorded data of surface characterization after the immersion test were statistically analyzed.

### 2.4. Frictional Force Evaluation

**Figure 1:**
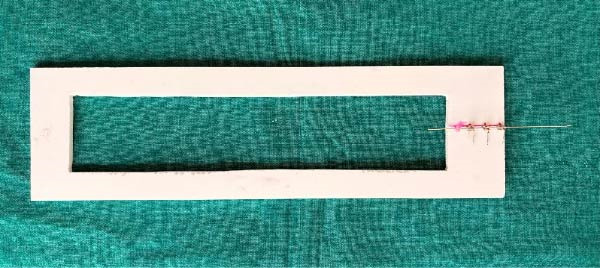
Bracket wire assembly on custom jig.

A total of 20 samples were included in the study, with 10 samples allocated to each group: Group A comprised uncoated SSWs, while Group B comprised coated SSWs. A custom‐made jig was designed for holding wires during testing, as shown in Figure 1. The brackets were bonded on the jigs, and ligation between the bracket and wire was with a SS ligature wire during frictional force evaluation, which was standardized with 11 turns per bracket. A universal testing machine was used to test the frictional force. A load cell was set up to read between 0 and 10 N, and the archwire was pulled through the bracket at a pace of 10 mm/min along a 5‐mm segment of the archwire. After each test, the bracket and wire were taken off, and a new set was put on. Statistical analysis was performed on the recorded data to discover whether there were any significant differences between the coated and untreated archwires.

## 3. Statistical Analysis

The data were examined with the statistical software SPSS Version 26.0 (SPSS Inc., Chicago, IL), with a significance threshold established at *p*  < 0.05. Descriptive statistics were employed to assess the mean and standard deviation of each group. Normality was assessed using the Shapiro–Wilk test. Intergroup comparison was conducted with the independent sample *t*‐test. Data were exhibited through tables and bar graphs as well as plot graphs.

## 4. Results

The comparison of the frictional force between Group A (uncoated) and Group B (coated) archwires under different loading conditions is presented in Table 1. Under normal load (N), Group A exhibited a higher mean frictional force of 11.31 ± 0.93, whereas Group B showed a significantly lower mean value of 6.39 ± 0.59. The difference between the two groups was found to be statistically highly significant (*p* < 0.001).

**Table 1:** Comparison of mean force (N) and stress (MPa) values between Group A (uncoated stainless‐steel wires) and Group B (coated stainless‐steel wires).

Parameter	Group A—uncoated		Group B—coated		*p*‐Value
	Mean	SD	Mean	SD	
N	11.31	0.93	6.39	0.59	<0.001
MPa	14.63	1.11	7.79	0.78	<0.001

Independent sample *t*‐test, *p* value < 0.05 is considered statistically significant, and SD—standard deviation.

**Figure 2:**
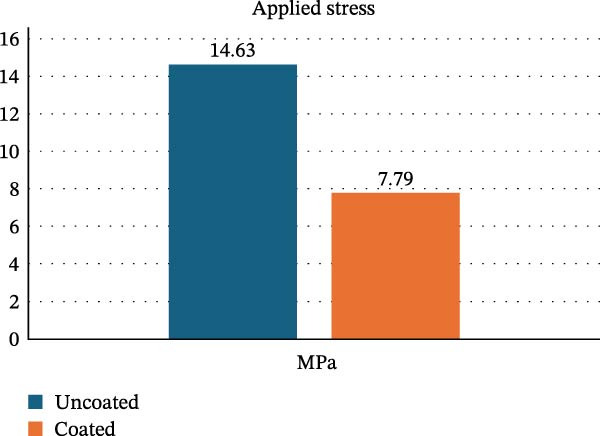
Comparison of applied stress (MPa) between uncoated stainless‐steel wires and coated stainless‐steel wires.

Figure 2 depicts the comparison of MPa between uncoated and coated archwires when subjected to a load. Group A demonstrated a mean MPa of 14.63 ± 1.11, while Group B again showed reduced MPa with a mean value of 7.79 ± 0.78. This difference was also statistically highly significant (*p* < 0.001).

The findings indicate that, even under increased applied stress, coated archwires maintain significantly lower resistance to sliding compared to uncoated archwires, suggesting improved surface characteristics and enhanced friction‐reducing properties of the coating. This indicates an approximate 46%–47% reduction in the frictional resistance in the coated group when compared to the uncoated group, highlighting the effectiveness of the surface coating in reducing friction during sliding mechanics. In both loading conditions, coated archwires consistently demonstrated lower frictional resistance compared to uncoated archwires. The observed differences in stress values between the groups reflect the reduced frictional resistance of the rGO‐coated archwires under identical testing conditions rather than differences in the applied load.

Raman spectroscopy confirmed the successful deposition of rGO on SS archwires, as given in Table 2. The presence of characteristic D (~1350 cm^−1^) and G (~1590 cm^−1^) bands in both rGO and rGO‐coated SSW (rGO‐SSW) samples indicates the retention of sp^2^ ‐bonded graphitic carbon following the coating process. In contrast, pure SSW exhibited weak or absent D and G bands, confirming that the observed Raman features originated from the rGO coating. The rGO sample showed a higher D‐band intensity and an ID/IG ratio of 1.30–1.40, reflecting a greater degree of structural disorder typical of chemically rGO. The rGO‐SSW samples demonstrated a slightly lower ID/IG ratio (1.20–1.30), suggesting reduced defect density and improved structural organization of rGO when deposited as a thin, uniform layer on the SS surface. This controlled defect‐rich yet graphetically ordered surface is favorable for orthodontic applications, as it may contribute to reduced surface irregularities and enhanced solid‐lubricant behavior, thereby lowering frictional resistance at the bracket–archwire interface. These Raman findings correlate with surface morphology observations and support the role of rGO coating in modifying the surface characteristics of SS archwires.

**Table 2:** Raman spectral parameters of rGO, rGO‐coated stainless‐steel wire, and pure stainless‐steel wire.

Sample	D band peak position (cm^−1^)	Width (cm^−1^)	Iᴅ (a.u.)	G band peak position (cm^−1^)	Width (cm^−1^)	Iɢ (a.u.)	Iᴅ/Iɢ
rGO	1350	70–80	1400–1500	1590	60–70	1000–1100	1.30–1.40
rGO‐SSW	1350	60	250–300	1590	55–60	200–250	1.20–1.30
SSW (pure substrate)	Weak/absent	—	~ 20–30	Weak/absent	—	20–40	0.5–0.7 Very low

**Figure 3:**
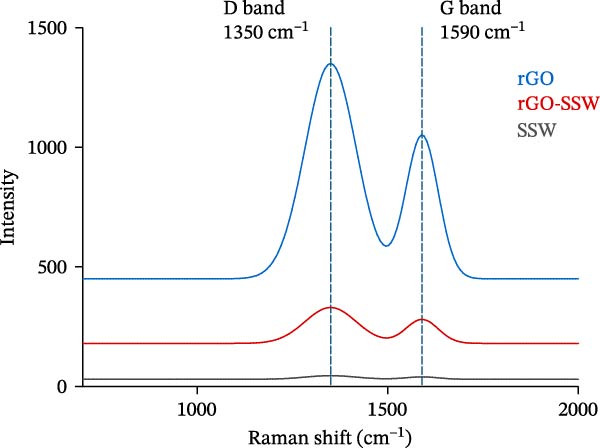
Raman spectra of reduced graphene oxide (rGO), rGO‐coated stainless‐steel wire (rGO‐SSW), and pure stainless‐steel wire (SSW).

Figure 3 shows the Raman analysis. The rGO‐coated SSW samples were characterized using Raman spectroscopy to confirm the successful deposition of graphene oxide. The typical Raman spectrum of rGO is characterized by a G band at 1590 cm^−1^, which corresponds to the E2g phonon of the sp^2^ C atoms, and a D band at 1319 cm^−1^, which corresponds to the breathing mode of k‐point phonons of A1g symmetry. Here, the G band of graphene was observed at ~1590 cm^−1^, corresponding to the in‐plane vibration of sp^2^ ‐bonded carbon atoms and confirming the graphitic nature of the deposited rGO layer. The D band is an indication of disorder, which may arise from certain defects such as vacancies, grain boundaries, and amorphous carbon species [15, 16]. The presence of these two bands in the rGO‐SSW sample indicates the coating of rGO.

**Figure 4:**
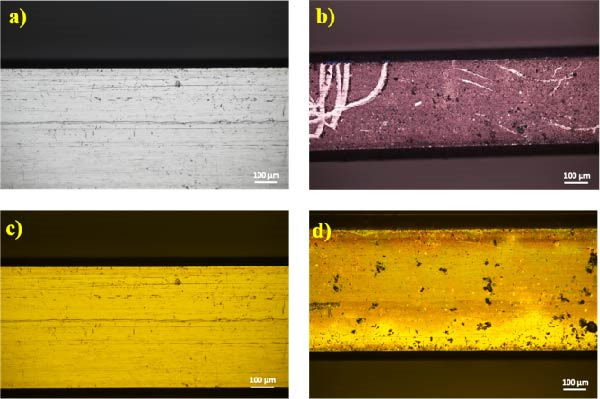
Optical microscope images of (a) and (c) bare SSW and (b) and (d) rGO‐coated SSW.

Optical microscopy images clearly reveal the distinction between the bare SSW and the rGO‐coated SSW, as seen in Figure 4. The coated wire exhibits a uniform surface layer compared to the uncoated sample. Additionally, the presence of intentional scratches on the coated surface makes the rGO layer more distinguishable, thereby confirming the successful coating.

**Figure 5:**
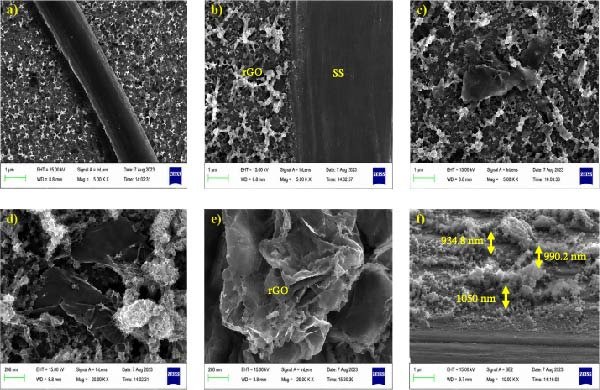
FESEM images of rGO‐coated samples at different magnifications showing surface morphology of the coated stainless‐steel wire: (a–f) surface morphology at increasing magnifications.

Figure 5 shows the FESEM images of rGO‐coated SSW. Further, the coating of rGO and the morphology were confirmed through FESEM images. The images clearly show the distinction between coated and noncoated surfaces. The magnified images show the presence of rGO sheets. The coating has a thickness of around 1 µm.

**Figure 6:**
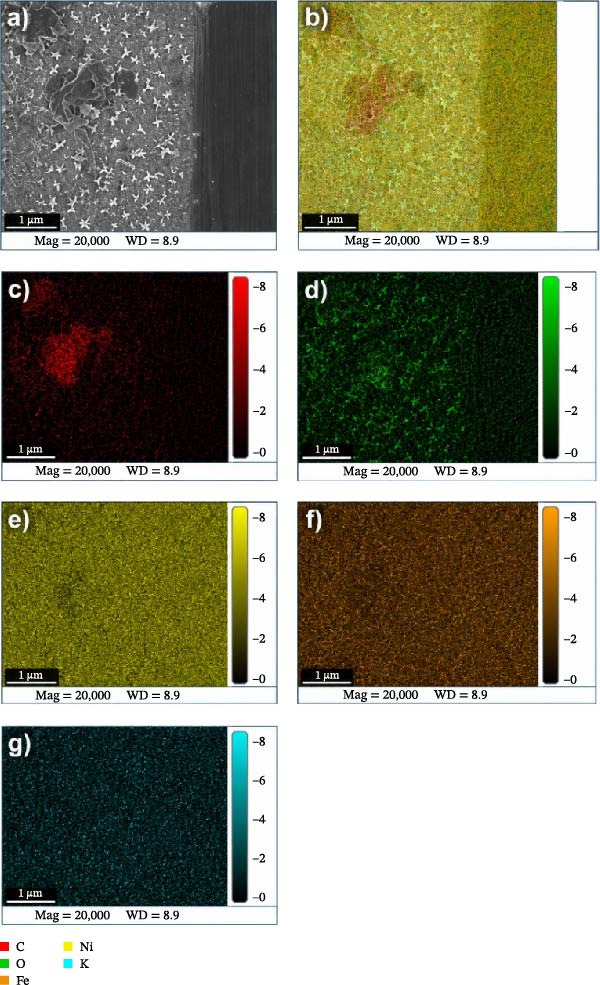
Elemental mapping of the rGO‐SSW sample showing the distribution of major elements across the coated surface: (a) SEM image, (b) carbon mapping, (c) oxygen mapping, (d) elemental overlay map, (e) iron mapping, (f) chromium mapping, (g) nickel mapping.

Figure 6 shows elemental mapping, which clearly reveals the distribution of carbon and oxygen over the coated surface, confirming the presence of rGO.

**Figure 7:**
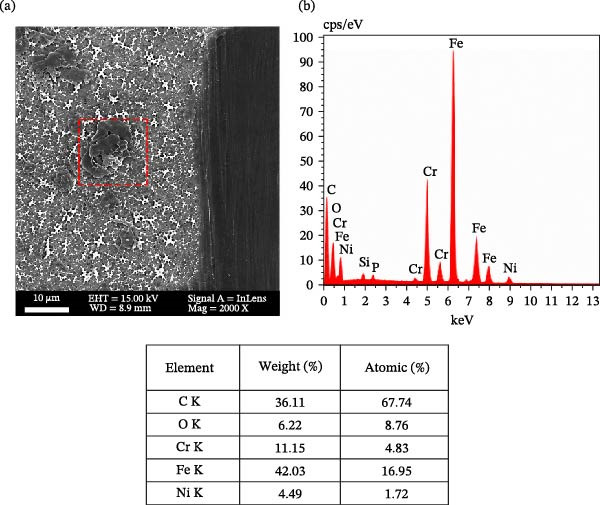
EDAX spectrum and elemental composition analysis of rGO‐coated stainless‐steel wire (rGO‐SSW): (a) EDAX spectrum and (b) corresponding elemental weight percentage distribution.

In addition, in Table 3 and Figure 7, EDX analysis validates the existence of these elements, thereby substantiating that the SSW surface is successfully coated with rGO.

**Table 3:** EDAX data of rGO‐SSW.

Element	Weight (%)	Atomic (%)
C K	36.11	67.74
O K	6.22	8.76
Cr K	11.15	4.83
Fe K	42.03	16.95
Ni K	4.49	1.72

## 5. Discussion

Frictional resistance at the bracket–archwire interface has long been recognized as a major biomechanical factor influencing the efficiency of orthodontic tooth movement [13]. SS archwires remain widely used in clinical orthodontics due to their favorable mechanical strength, dimensional stability, and corrosion resistance. However, the microstructural surface irregularities inherent to SS contribute to increased frictional resistance through mechanical interlocking within bracket slots. Under increased loading conditions, such as those encountered during space closure or en masse retraction, friction tends to escalate due to enhanced contact pressure and deformation at the interface [17].

In tribological science, a reduction of friction is commonly achieved through surface modification, lubrication, or material substitution. In orthodontics, surface engineering strategies have gained attention as a means of improving the sliding efficiency without compromising the intrinsic mechanical properties of archwires [17].

In recent years, graphene and its derivatives have emerged as promising nanomaterials in biomedical and tribological applications. Its lamellar structure allows adjacent layers to slide over one another with minimal resistance due to weak interlayer van der Waals forces [14]. It aids in exhibiting exceptional mechanical strength, chemical stability, and inherent solid‐lubricant properties.

rGO, obtained by the chemical reduction of graphene oxide, retains the layered architecture of graphene while providing enhanced adhesion and processability. Tribological studies conducted on metallic substrates coated with graphene derivatives have demonstrated substantial reductions in the friction coefficients and wear rates. In orthodontic applications specifically, graphene‐coated nickel–titanium and SS archwires have shown improved sliding performance and enhanced wear resistance in artificial saliva environments [18].

Although previous studies suggest that graphene‐based coatings can reduce friction, several limitations remain in the existing literature. Most investigations have focused on nickel–titanium alloys rather than SS archwires, which continue to be extensively used in clinical orthodontics [15, 18].

Furthermore, limited data exist regarding the structural organization of rGO when deposited on orthodontic substrates and how such structural characteristics correlate with tribological performance. In particular, the relationship between Raman spectroscopic parameters, such as the Iᴅ/Iɢ ratio, and frictional behavior in orthodontic systems has not been comprehensively explored. These gaps in knowledge provided the scientific rationale for the present investigation.

The first finding of our study is the statistically significant reduction in the frictional force observed in the coated group, highlighting the ability of rGO to function as an effective solid lubricant. This finding of the present study is in accordance with the results reported by Pan et al., where significant reductions in friction coefficients were observed following graphene‐based surface modification [19]. Similarly, the present investigation demonstrated a statistically significant reduction in frictional resistance in rGO‐coated SS archwires compared to uncoated controls under both normal load and stress conditions.

Graphene‐based materials are characterized by a two‐dimensional lamellar structure composed of sp^2^‐bonded carbon atoms arranged in hexagonal lattices. This has also been concluded by Kwon et al. [20], who examined the influence of thickness and chemical reduction of graphene oxide on frictional behavior. Their study demonstrated that rGO exhibited significantly lower friction compared to graphene oxide due to the restoration of the sp^2^ carbon lattice. The weak van der Waals forces between adjacent layers allow easy interlayer sliding, thereby reducing the resistance to motion. When applied as a surface coating, rGO forms a thin lubricating layer that facilitates smoother sliding at the bracket–archwire interface [20].

Achieving a uniform and defect‐free rGO coating remains challenging because graphene sheets tend to agglomerate during deposition and may form wrinkles, folds, and microstructural defects. Parameters such as suspension concentration, deposition time, applied voltage, and substrate preparation critically influence coating uniformity.

Another contributing factor to reduced friction is the modification of the surface topography. SS archwires typically exhibit microscale surface irregularities that promote mechanical interlocking with bracket slots. The deposition of rGO tends to fill and mask these asperities, resulting in a more uniform and smoother surface. This reduction in surface roughness minimizes mechanical interlocking and contributes to lower frictional resistance during sliding.

The magnitude of friction reduction observed in this study is clinically relevant as even modest reductions in friction can significantly influence the rate and predictability of orthodontic tooth movement. The consistent reduction in friction under normal load suggests that rGO‐coated archwires may be particularly beneficial during early alignment and leveling stages, where controlled and efficient sliding mechanics are critical.

In the present study, both uncoated and coated archwires exhibited an increase in the frictional force when subjected to increased stress. However, the magnitude of this increase was significantly lower in the rGO‐coated group.

The ability of rGO‐coated archwires to maintain relatively low frictional resistance under increased load conditions indicates that the coating remains mechanically stable and functionally effective even under higher stress. This behavior can be attributed to the high mechanical strength and flexibility of graphene‐based materials. rGO exhibits excellent load‐bearing capacity while maintaining its lubricating properties, thereby preventing direct metal‐to‐metal contact even under increased pressure [19].

Furthermore, graphene‐based coatings are known to form transfer films or tribofilms during sliding contact. These tribofilms act as protective layers that further reduce friction and wear by distributing contact stresses more evenly across the surface. The formation of such films is particularly advantageous in wet environments, such as the oral cavity, where saliva may interact synergistically with graphene‐based surfaces to enhance lubrication.

From a clinical standpoint, the sustained reduction in friction under increased load conditions suggests that rGO‐coated archwires could improve treatment efficiency during the mechanically demanding stages of orthodontic therapy. This may allow clinicians to achieve desired tooth movements with lower applied forces, potentially reducing treatment duration and improving patient comfort.

Raman spectroscopy is a widely accepted and reliable technique for characterizing graphene‐based materials due to its sensitivity to carbon bonding configurations and structural orders.

The surface characterization findings of the present study are consistent with both the nanoscale tribological analysis reported by Kwon et al. [20] and the orthodontic coating investigation conducted by Dai et al. [21]. Kwon et al. [20] examined the effect of chemical reduction of graphene oxide on nanoscale friction and demonstrated that rGO exhibits distinct Raman signatures characterized by prominent D (~1350 cm^−1^) and G (~1580–1600 cm^−1^) bands [17]. They further emphasized that the Iᴅ/Iɢ ratio serves as a critical indicator of defect density and structural restoration within the graphene lattice [22].

In the present study, Raman analysis revealed the presence of prominent D and G bands at ~1350 cm^−1^ and 1590 cm^−1^, respectively, in both rGO powder and rGO‐coated SS archwire samples.

The G band corresponds to the in‐plane vibration of sp^2^‐bonded carbon atoms and is indicative of graphitic structures, while the D band is associated with defects, disorder, and edge states within the graphene lattice. The absence or negligible presence of these bands in uncoated SSWs confirms that the detected Raman signals in coated samples originated exclusively from the rGO layer [17].

The intensity ratio of the D band to the G band (Iᴅ/Iɢ) provides valuable insights into the degree of disorder within the graphene structure. In this study, rGO powder exhibited a relatively higher Iᴅ/Iɢ ratio, reflecting increased defect density resulting from chemical reduction processes. Interestingly, the rGO‐coated SSWs demonstrated a slightly lower Iᴅ/Iɢ ratio, suggesting improved structural organization when rGO was deposited as a thin coating [19].

This reduction in defect density upon coating may be attributed to the alignment and redistribution of graphene sheets on the metallic substrate. A moderately defect‐rich yet structurally organized graphene layer is considered ideal for tribological applications as defects enhance adhesion to the substrate while excessive disorder may increase surface roughness and friction. The Raman findings of this study indicate that the rGO coating achieved an optimal balance between adhesion and lubricating efficiency.

The frictional behavior observed in this study correlates well with the Raman spectroscopic findings [19]. The presence of a well‐adhered, structurally organized rGO layer on the SS surface is likely responsible for the significant reduction in frictional resistance. The graphene‐based coating modifies surface chemistry, topography, and interfacial mechanics synergistically, resulting in improved sliding performance.

Reduced defect density and improved graphitic order facilitate easier interlayer shear, which is essential for effective solid lubrication. At the same time, sufficient defect sites ensure strong adhesion of the coating to the substrate, preventing delamination during mechanical loading. This balance between structural order and defect‐mediated adhesion is critical for maintaining consistent tribological performance over time [23].

The findings of this study reinforce the concept that surface modification at the nanoscale can significantly influence macroscopic mechanical behavior, particularly in orthodontic applications, where sliding mechanics play a central role.

The results of the present investigation agree with previously reported studies evaluating graphene‐based coatings in orthodontics and dental biomaterials, as stated by Liu et al. [24].

In addition to friction reduction, previous studies have demonstrated that graphene‐based coatings can enhance the corrosion resistance, wear resistance, and antibacterial activity of orthodontic materials. While the present study focused primarily on frictional behavior and surface characterization, the findings align with the broader consensus that graphene‐based surface modifications offer multifunctional benefits. The consistency between the present findings and the existing literature strengthens the validity of rGO as a promising surface coating for orthodontic archwires.

The clinical implications of the present study are significant. Reduced friction at the bracket–archwire interface can enhance the efficiency of orthodontic tooth movement, allowing for better force control and potentially shorter treatment durations. Lower friction may also reduce the need for excessive force application, thereby minimizing patient discomfort and reducing the risk of adverse biological effects. Previous studies have shown that graphene‐based coatings may undergo gradual degradation in the oral environment because of pH fluctuations, enzymatic activity, and repeated mechanical loading. However, certain graphene coatings have demonstrated good chemical stability and wear resistance under simulated oral conditions, suggesting potential for long‐term applications[25].

The ability of rGO‐coated archwires to maintain low friction under increased load conditions suggests their suitability across various stages of the orthodontic treatment. Furthermore, the nanoscale thickness of the rGO coating ensures that the bracket slot dimensions and wire stiffness remain largely unaffected, preserving the biomechanical integrity of the appliance system.

## 6. Limitations of the Study

The in vitro design of this study limits direct application to clinical circumstances despite the promising results. Salivary enzymes, temperature swings, and extended mechanical fatigue are examples of dynamic elements found in the oral environment. The study did not evaluate long‐term coating stability, corrosion resistance, cyclic mechanical loading, or biocompatibility. Future studies should optimize deposition parameters to achieve smoother and more homogeneous coatings, evaluate long‐term coating stability under simulated oral conditions, investigate antibacterial properties and corrosion resistance, and perform in vivo clinical studies.

## 7. Conclusion

The use of rGO coating markedly decreases the frictional resistance of SS orthodontic archwires in comparison to uncoated wires. This study combines tribological assessment with structural characterization to offer both mechanical and molecular validation of rGO coating efficacy.

## Author Contributions

**P. Shreeya Varma**: conceptualization, methodology, investigation, data curation, formal analysis, writing – original draft. **Arun S. Urala**: conceptualization, methodology, supervision, writing – review and editing. **Ramya Vijeta Jathanna**: methodology, formal analysis, writing – review and editing, supervision, correspondence. **Suresh D. Kulkarni**: investigation, validation, resources, writing – review and editing.

## Funding

No external funding was received for this manuscipt.

## Disclosure

All authors have read and approved the final version of the manuscript. Ramya Vijeta Jathanna had full access to all of the data in this study and takes complete responsibility for the integrity of the data and the accuracy of the data analysis.

## Ethics Statement

This in vitro study did not involve human participants or animals. Ethical approval was obtained from the Institutional Ethics Committee of Kasturba Medical College and Kasturba Hospital (IEC2: 336/2024).

## Conflicts of Interest

The authors declare no conflicts of interest.

## Data Availability

The data are available upon request due to privacy/ethical restrictions.
